# A Perspective on Objective Measurement of the Perceived Challenge of Walking

**DOI:** 10.3389/fnhum.2019.00161

**Published:** 2019-05-14

**Authors:** Sudeshna A. Chatterjee, Dorian K. Rose, Eric C. Porges, Dana M. Otzel, David J. Clark

**Affiliations:** ^1^Brain Rehabilitation Research Center, Malcom Randall VA Medical Center, Gainesville, FL, United States; ^2^Department of Physical Therapy, University of Florida, Gainesville, FL, United States; ^3^Department of Clinical and Health Psychology, University of Florida, Gainesville, FL, United States; ^4^Department of Aging and Geriatric Research, University of Florida, Gainesville, FL, United States

**Keywords:** walking, galvanic skin response, rehabilitation, gait, physiological stress response

## Abstract

Perceived challenge of walking is a broad term that we use to encompass walking-related anxiety, balance self-efficacy/confidence, and fear of falling. Evidence shows that even after accounting for physical performance capabilities, a higher perceived challenge can cause individuals to self-impose restrictions in walking-related activities. Perceived challenge is typically measured by self-report, which is susceptible to subjective measurement bias and error. We assert that measurement of perceived challenge can be enhanced by augmenting self-report with objective, physiologically based measures. A promising approach that has emerged in the literature is measurement of sympathetic nervous system (SNS) activity by recording skin conductance. Heightened SNS activity is a physiological stress response to conditions that are cognitively, emotionally, or physically challenging. In the present article, we explain the rationale and physiological basis for measuring SNS activity to assess perceived challenge of walking. We also present existing and new evidence supporting the feasibility of this approach for assessing perceived challenge in lab-based and real-world walking environments. Future research directions are also discussed.

## A New Perspective on Quantifying Walking Function: Objective Measurement of Perceived Challenge

For people with neurological impairment or injury, participation in life-role activities is often restricted by walking function. Preserving or restoring participation, therefore, requires a thorough understanding of underlying factors that influence walking function. Physical impairments are known to be an influential factor, but emerging research shows that an individual’s own perceived challenge of walking may be equally, if not more, influential. Perceived challenge of walking is a broad term that we use to encompass walking-related anxiety, balance self-efficacy/confidence, and fear of falling. Evidence shows that even after accounting for physical performance capabilities, a higher perceived challenge can cause individuals to self-impose restrictions in walking-related activities. Given the apparent importance of perceived challenge for walking function and participation, increased scrutiny is warranted regarding how this construct is measured. Perceived challenge is typically measured by self-report, which is susceptible to subjective measurement bias and error. In the present article, we offer a new perspective on an objective, physiologically-based measurement based on recording activity from the sympathetic nervous system (SNS). In the next several sections we will: (1) review the relationship between higher perceived challenge of walking and participation in walking-related activities; (2) describe the limitations of self-report as an indicator of perceived challenge; (3) present the rationale for using SNS activity as a neurophysiological measure of perceived challenge; (4) discuss evidence supporting the feasibility of measuring SNS activity during community walking; and (5) propose future directions for this area of research.

## Perceived Challenge of Walking and Participation in Walking-Related Activities

Higher perceived challenge of walking contributes to a reduction in walking activity and participation, even after accounting for physical performance capabilities (Tinetti and Powell, 1993; Franzoni et al., 1994; Robinson et al., 2011). In a study examining factors associated with post-stroke participation in community walking, Robinson et al. (2011) reported that self-efficacy related to balance and falls was the only personal characteristic that was strongly associated with both subjective and objective measures of participation in community walking. They reported that higher self-efficacy was associated with lower perceived difficulty of walking (measured by the modified Mobility and Self-Care questionnaire; Farin et al., 2007) and greater engagement in walking-related activities and participation in community walking (measured by a trip activity log). Ehlers et al. (2017) reported that in older adults, gait self-efficacy (measured by the Gait Self-Efficacy Scale; McAuley et al., 1997) and not measures of physical function (lower-extremity mobility, endurance, and strength) significantly predicted the performance on real-world simulated walking (street-crossing) using virtual reality. Similarly, Danks et al. (2016) reported that balance self-efficacy significantly predicted engagement in daily walking activity post-stroke even after accounting for the metabolic cost of walking, postural stability, depression, fatigue, and self-reported health status. Likewise, Schmid et al. (2012) reported that balance self-efficacy and not physical aspects of gait (walking speed and walking capacity/distance walked) was independently associated with both activity and participation post-stroke (measured by ICF Measure of Participation and Activities; Post et al., 2008).

The relationship between perceived challenge and participation in walking activities is likely more prominent when walking in environments with complex task demands. Examples of complex tasks include those requiring obstacle negotiation, uneven surfaces, distractions, and/or multi-tasking (Patla and Shumway-Cook, 1999). Acute feelings of task-related anxiety, fear of falling and low balance/falls self-efficacy have been consistently reported during performance of complex walking tasks (Brown et al., 2002; Hadjistavropoulos et al., 2012). The community environment imposes a variety of tasks with heightened physical and cognitive demands, which pose differing levels of challenge to the individual based on his/her own abilities. This interplay between the environment and the individual will affect perceived challenge, and in turn the likelihood that the person may engage in or avoid participation.

## Limitations of Self-Report for Assessing Perceived Challenge

Measures of self-report are clearly valuable, as they have yielded aforementioned knowledge about the link between perceived challenge and participation. Self-report measures are widely used in research because they are standardized, mostly inexpensive, easy to administer, and time-efficient. However, the self-report method is also susceptible to several forms of measurement bias (Orne, 1962; Donaldson and Grant-Vallone, 2002; Paulhus and Vazire, 2007). Demand characteristics (Orne, 1962) are a common form of response bias in which participants subconsciously alter their responses to fit their interpretation of the study purpose or adopt certain behaviors that they believe would please the investigator or benefit the study. Social desirability bias occurs when a participant reports only behaviors that they feel are socially acceptable. Acquiescence bias is the tendency to agree with all statements, even when it results in contradictory responses. Extreme response style bias is the tendency to select only the extreme endpoints of a rating scale. Furthermore, rating scales may have limited or imprecise response choices that do not adequately match the individual’s feelings. Bias or error can also occur when there are individual differences in language proficiency, cultural norms, levels of literacy, personality traits, or introspective ability. While self-report remains a valuable approach for assessing perceived challenge, there is justification for supplementing this information with objective physiological measures.

## Sympathetic Nervous System Activity Measured by Skin Conductance as an Objective Physiological Measure of Perceived Challenge

The SNS is the part of the autonomic nervous system that elicits the “fight or flight” response. It is activated by acute states of increased attention, stress, and/or anxiety (Adelman et al., 1975; Critchley, 2002; Boucsein, 2012). SNS response is increased during physically or cognitively challenging situations in order to biologically and behaviorally prepare an individual for action. Increased SNS activity upregulates the release of stress hormones (e.g., epinephrine, norepinephrine, cortisol), increases cardiopulmonary responses, decreases digestive processes, diverts blood flow towards the working muscles, and increases sweating. While most of these responses are controlled by both the sympathetic and parasympathetic divisions of the autonomic nervous system, sweating is controlled primarily by the SNS. For this reason, change in skin conductance (also known as galvanic skin response or electrodermal response) due to emotion-induced sweating is a widely accepted non-invasive approach for measuring SNS activation, as discussed in the 2012 committee report from the Society for Psychological Research *Ad Hoc* Committee on Electrodermal Measures (Boucsein et al., 2012; Boucsein, 2012). Increased sweat gland activity decreases the skin’s resistance to the flow of electricity, which is measured as increased conductance when a weak electrical current is applied to the skin. SNS induced changes in skin conductance are generally measured from the palmar surface of the hand, because it contains eccrine sweat glands that are particularly responsive to emotion-induced cholinergic stimulation of the sudomotor nerves (Boucsein et al., 2012; Boucsein, 2012; van Dooren et al., 2012). Thermoregulatory processes have only a minimal influence on palmar sweating, so this site is ideal for gauging emotional stress-related responses (Kerassidis, 1994).

The two commonly analyzed components of the skin conductance signal are skin conductance level (SCL), and skin conductance response (SCR; Benedek and Kaernbach, 2010; Figner and Murphy, 2011; Boucsein, 2012). SCL is an indicator of the changes in skin conductance amplitude over time periods of several seconds or longer and can be considered a global or “background” measure of changes in SNS activity. SCR is an indicator of high frequency changes in the skin conductance signal that are thought to more closely represent the temporal activity of the sudomotor nerves.

Several studies have successfully demonstrated that SNS activity, measured by skin conductance, is robustly increased during complex tasks. The magnitude of change in SNS activity is associated with a greater perceived challenge, even when actual task demands remain constant. For instance, Adkin et al. (2002) measured skin conductance to assess the SNS response to postural instability and fear of falling and reported a 63.5% increase in SCL when the participants were asked to rise to toes on the edge of an elevated platform (vs. at a lower level). The authors reported that the increase in skin conductance was in agreement with self-reported lower levels of balance confidence and stability, and higher levels of self-reported anxiety. Similar findings have also been reported by Hadjistavropoulos et al. (2012) who measured SNS response to task-related anxiety/fear of falling during walking on surfaces of varying heights, as well as during dual-task walking. The authors reported a significant increase in skin conductance during dual-task walking with a tray on an elevated platform which is consistent with a greater task-related threat to postural stability and increased feelings of anxiety and fear of falling during the task performance.

Our research group (Clark et al., 2014) has reported increased magnitude of SNS activity in older adults during the preparation (prior to initiating movement) and the performance phase of several complex walking tasks including cognitive and motor dual-task walking, and obstacles crossing. Compared to the preparation phase of typical walking, SNS activity during complex walking tasks was significantly greater during both the preparation phase (task anticipation) and performance phase. Furthermore, for each complex walking task, SNS activity increased from the preparation phase to the performance phase. This is consistent with a greater perceived challenge when actually performing a complex walking task vs. just anticipating task performance.

We have also reported skin conductance measurement of SNS response to complex walking in adults with chronic post-stroke hemiparesis. In this population, higher magnitude of SNS activity measured by skin conductance was shown to be associated with worse task performance across 17 walking tasks, as quantified by speed of movement or performance grading by a physical therapist (Clark et al., 2018). This finding supports that SNS activity scales with the challenge level of the task. Furthermore, we found that individuals with poorer mobility function exhibited significantly higher SNS activity during the performance of the walking tasks, particularly those that were the most difficult. This is consistent with a greater perceived challenge of walking for individuals who are more impaired. In a separate study, we showed that the SNS response was significantly higher during the performance of complex tasks such as obstacles crossing and cognitive dual-task walking compared to typical walking (Chatterjee et al., 2018). Furthermore, the SNS response to complex walking tasks was reduced significantly following a 12-week walking rehabilitation program. Importantly, the intervention-induced reduction in SNS response was accompanied by a significant increase in self-reported balance self-efficacy measured by the Activities-specific Balance Confidence (ABC) Scale (Powell and Myers, 1995). Cumulatively, the published work reviewed here supports that SNS activity measured by skin conductance is a useful and feasible approach for measuring the perceived challenge of walking in people with high or low walking function.

## Feasibility of SNS Measurement in Community Walking Environments

Skin conductance can be measured continuously in either lab-based or real-world settings by wearable sensors that are unobtrusive and inexpensive. This opens up many possibilities for assessing the perceived challenge of walking under ecologically valid conditions, which is important for understanding the link to participation in life-role activities. However, there are potential challenges to implementing this approach in “real-world” community environments that are less controlled and predictable than a research laboratory. Many factors unrelated to walking can introduce variability to the skin conductance data, such as when participants attend to unrelated distractions in the environment (e.g., nearby people or objects) or encounter different ambient conditions such as noisy surroundings or differing temperature. These factors can differ both across days of testing as well as within a given testing session as participants move through different parts of the environment. A potential concern is that given the unpredictable and attentionally arousing nature of real-world environments; the “signal to noise” ratio of the skin conductance recordings may be compromised, making it harder to detect walking task-induced changes in the measurement. Therefore, we conducted a pilot study to establish feasibility for detecting walking task-related changes in SNS activity in adults’ post-stroke during walking in a real-world community setting. Eight adults with chronic post-stroke hemiparesis (age = 63.1 ± 9.9 years; chronicity = 68.1 ± 54.6 months; see Table 1A for clinical data) and eight healthy young adults (age = 22.4 ± 3.7 years) performed complex walking tasks in the publicly accessible areas of a major hospital. The complex tasks were based on the domains essential for community ambulation (see Table 1B for description of tasks). Immediately after completion of each task, the participants were asked to rate their balance confidence using the stem question, “On a scale of 0%–100%, how confident were you that you would not lose your balance or become unsteady while you performed the task?” (Powell and Myers, 1995).

**Table 1A:** Clinical assessments for the post-stroke group.

Clinical assessments	
10 MWT (m/s)	0.6 ± 0.2
ABC Scale (%)	75.3 ± 12.3
STAI (State component out of 80)	22.8 ± 4.7
STAI (Trait component out of 80)	26.3 ± 9.5
DGI (out of 24)	16.0 ± 3.6
SF-36 Physical functioning (out of 100)	64.8 ± 17.9
SF-36 Role limitations due to physical health (out of 100)	43.7 ± 47.7

*Abbreviations: 10 MWT, 10 Meter Walk Test; ABC Scale, Activities-specific Balance Confidence Scale; STAI, State-Trait Anxiety Inventory; DGI, Dynamic Gait index; SF-36, Short Form Health Survey. The errors denote standard deviation*.

**Table 1B:** Description of the walking tasks in the community ambulation assessment.

Walking task	Domain(s)*	Description	
Ramp	Terrain	The participants walked along an outdoor ramp (mild decline then moderate incline) through a busy and noisy loading dock area.
Cafeteria	Maneuvering in traffic Cognitive dual-tasking	The cafeteria is a busy and noisy space with many people walking about in irregular paths. Participants were instructed to enter the cafeteria, use overhead menu boards to determine the price of a hamburger and the price of a coffee, then exit the cafeteria.
Convenience store	Maneuvering in traffic Cognitive dual-tasking Postural transitions	The convenience store is a relatively busy space with narrow aisles. It was common for participants to walk in close proximity to other shoppers and around racks/shelves, and to bend down to view shelves of different heights. The participants were instructed to locate soft drinks and paper towels.
Elevator	Cognitive dual-tasking	The participants were instructed to walk down a hallway and follow signs to the elevator, then to push the buttons necessary to call the elevator and take it to the 2nd floor. They then exited the elevator and proceeded to walk a short distance down the hallway.
Indoor stairs	Terrain	The participants were instructed to open the door to a stairwell, descend one flight of stairs, and open another door to exit the stairwell.
Outdoor stairs	Terrain	The participants walked briefly on an outdoor covered walkway then descended a flight of stairs.
Grass	Terrain	The participants walked for approximately 20 m on grass.
Conversation	Cognitive dual-tasking	The participants walked on an outdoor sidewalk while engaged in casual conversation with a staff member. Standardized questions with relatively neutral content were used to guide the conversation, such as the location of the participant’s home, how many years he/she has lived in the area and his/her commute to the study site.
Dim lighting	Ambient conditions	The participants walked about 50 m on a designated path in a large darkened room. The lights were turned off, but some light was allowed to come in through the door of an adjacent room.

*Domains are based on prior published work (Patla and Shumway-Cook, 1999; Balasubramanian et al., 2014).

### Skin Conductance Data Recording and Analysis

Task-related SNS response was measured by skin conductance (SCL and SCR), and the data were analyzed in accordance with our previously published work (Chatterjee et al., 2018; Clark et al., 2018). Skin conductance signals were recorded using a commercially available portable data acquisition unit (Flexcomp Infiniti; Thought Technologies Ltd, Montreal, QC, Canada). Adhesive electrodes (10-mm Ag/AgCl recording surface) with conductive paste (0.5% saline in a neutral base) were placed on the palmar surface of the proximal phalanges of the index and ring fingers.

Data analysis was conducted with Matlab version R2011b (The Mathworks, Natick, MA, USA) using Ledalab v3.4.7 and custom analysis programs. The raw skin conductance data were down-sampled to 8 Hz and visually examined for the presence of signal artifact. Relatively few artifacts were identified, and the ones that were found were removed and replaced by linear interpolation. Continuous decomposition analysis was performed in Ledalab v3.4.7 to separate the tonic component (SCL) and the phasic component (SCR) of the signal. A minimum amplitude criterion of 0.04 microsiemens (μS). was applied to achieve a balance between sensitive detection of SCRs and minimizing the effects of movement artifacts (Benedek and Kaernbach, 2010).

**Figure 1:**
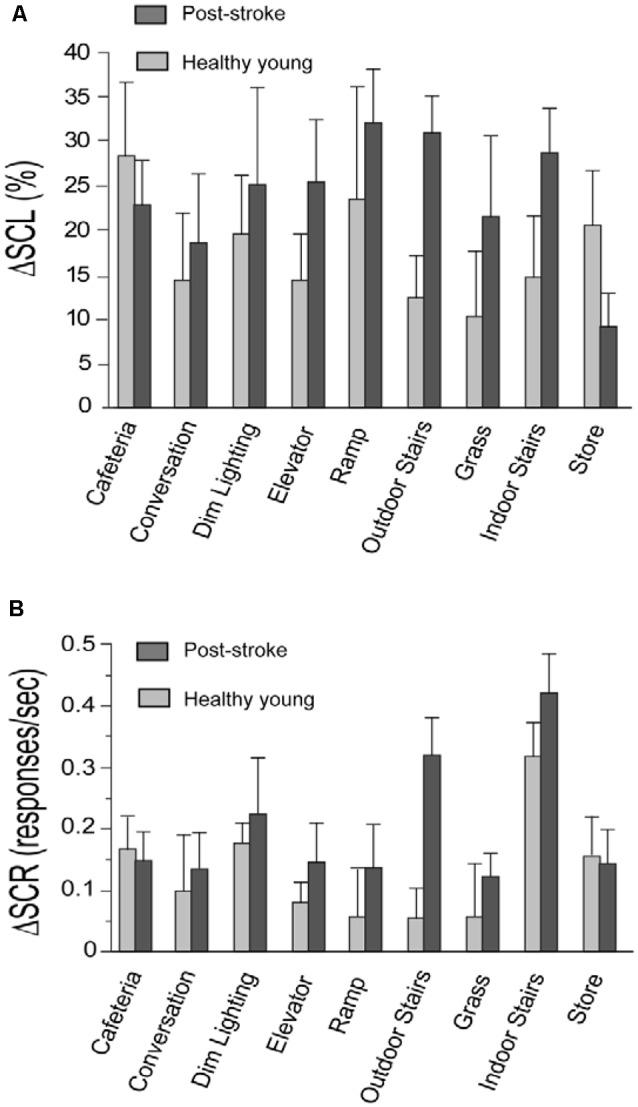
Change in skin conductance level (ΔSCL) and skin conductance response (ΔSCR) for the stroke and healthy young groups. Panels **(A,B)** show the change in skin conductance (i.e., ΔSCL and ΔSCR, respectively) from the resting to the walking period of each task. The stroke group is shown in dark gray and the healthy young group is shown in light gray. The error bars denote the standard error.

For each walking task, two values were extracted from SCL data. The first value was the minimum SCL that occurred during the seated resting period that preceded the walking task. The second value was the median SCL occurring during the duration of the walking task. The percent change in SCL (denoted by ΔSCL; Figure 1A) between the resting and walking period of each task was calculated using the following formula:

$$\text{ΔSCL = }\left[\frac{\left(\text{Walking Median}-\text{Resting Minimum}\right)}{\text{Resting Minimum}}\right]*100$$

For each walking task, two values were extracted from SCR data. The first value was the rate of SCRs occurring during the duration of the seated resting period that preceded the walking task. The second value was the rate of SCRs occurring during the walking task. The rate of SCRs refers to the number of SCRs divided by the duration of the recording period for each task. The change in the rate of SCR (denoted by ΔSCR; Figure 1B) from the resting to the walking period of each task was calculated using the following formula:

ΔSCR = (Rate of SCR during walking−Rate of SCR during rest)

### Statistical Analysis

Statistical analysis of the skin conductance data were conducted using JMP software (JMP^®^ 11. Cary, NC: SAS Institute Inc.). Within each group, paired *t*-tests were used to assess differences between the resting and walking periods of each task for SCL and SCR. The false discovery rate (FDR) procedure was used to correct for multiple comparisons (Curran-Everett, 2000; Curran-Everett et al., 2004). Pearson product-moment correlation coefficient was conducted to assess the strength of the correlation between the skin conductance measures (i.e., ΔSCL and ΔSCR) acquired from each hand.

## Results

In support of our study objective, we found that both components of the skin conductance signal (SCL and SCR) were increased in response to walking tasks performed in the community setting (Figure 1). This supports that the task-related changes in SNS activity are larger than SNS activity induced by measurement noise from the distractions and unpredictable conditions in the community setting. Consistent with our prior lab-based studies, we observed very few artifacts in the recorded signals and there was a strong correlation between skin conductance data recorded separately from each hand for the stroke group (ΔSCL, *r* = 0.82, *p* < 0.001; ΔSCR, *r* = 0.47, *p* < 0.001) and the young healthy group (ΔSCL, *r* = 0.92, *p* < 0.001; ΔSCR, *r* = 0.93, *p* < 0.001). This is supportive of high-quality skin conductance data with changes that are induced centrally by the SNS.

For both the post-stroke and young healthy groups, SCL was higher during walking compared to rest for all tasks (*p* ≤ 0.03, all significant after FDR; Figure 1A). The rate of SCR (responses/second) during walking was also higher compared to the resting period for all the tasks in the post-stroke group (*p* ≤ 0.04, all significant after FDR; Figure 1B). In contrast, healthy young adults exhibited higher SCR during walking for only a subset of the tasks (*p* ≤ 0.02, significant after FDR for Indoor Stairs, Dim Lighting, Cafeteria, Store, and Elevator). This is consistent with the expectation that walking is more challenging for individuals after a stroke compared to healthy individuals without neurological injury. A higher rate of SCRs during walking may be triggered acutely by poor movement control and greater episodes of unsteadiness/task-related stress after stroke. In comparison, younger adults have better movement control and are likely to experience fewer stress-provoking events such as due to postural instability. In addition, the absence of a change in SCRs between resting and walking for some tasks supports that SCRs are not an artifact of movement, but rather are likely due to task-related stress.

We observed that post-stroke balance confidence scores were among the lowest for the stairs negotiation tasks (Table 1C). Consistent with this observation, we also noted a significant increase in skin conductance measures (SCL and SCR) during the performance of the stairs tasks, compared to the resting period. These observations align with the known higher demands of stair negotiation relative to walking on a flat surface, as well as the potentially more severe consequences if a fall was to occur (Hamel and Cavanagh, 2004). This finding is also consistent with prior reports that negotiation of stairs is particularly challenging for older adults (Startzell et al., 2000). Lower self-efficacy/balance confidence when walking on stairs is associated with fear of falling, increased risk for falls, and mobility disability due to fear-avoidance of negotiating stairs (Shumway-Cook et al., 2002).

**Table 1C:** Post-stroke self-reported balance confidence after the completion of community ambulation tasks.

Walking task	Confidence level (%)
Grass	80 ± 13.2
Indoor stairs	80 ± 18.3
Outdoor stairs	85 ± 11.6
Conversation	85 ± 7.0
Ramp	93 ± 7.0
Convenience store	93 ± 7.0
Elevator	93 ± 10.3
Dim lighting	95 ± 12.2
Cafeteria	97 ± 5.1

## Conclusions and Future Directions for Research

A higher perceived challenge of walking can significantly impact the decision to engage in or avoid participation. Therefore, an accurate assessment of perceived challenge is vital for preserving participation in walking-related activities, maintaining independence, and fulfillment of life-roles, especially in populations vulnerable to mobility restrictions. There is strong evidence supporting the validity and feasibility of measuring the perceived challenge of walking using skin conductance measurement of SNS activity. This measurement approach has been successfully applied in both healthy and neurologically injured (i.e., post-stroke) research participants.

There are several potential directions for future research in this area. Our prior work (Chatterjee et al., 2018; Clark et al., 2018) supports the measurement of skin conductance to assess the perceived challenge of walking, which could be expanded to assess prediction of future adverse mobility outcomes such as falls. We have also shown evidence of a rehabilitation induced reduction of SNS activity during complex walking. Future research could further establish SNS activity as a biomarker of recovery, and as an outcome for comparing efficacy across multiple types of interventions. Future research could also add to the existing evidence of an association between self-report and the perceived challenge of walking. Prior evidence showing that skin conductance can be used to categorize the difficulty level of tasks (Clark et al., 2018) might be extended to the development of an ecologically valid walking task battery for the assessment of complex walking impairment. Another promising topic may be the measurement of skin conductance in real-time to optimize task challenge level during rehabilitation. Evidence shows that motor learning of a task is optimized when the level of challenge experienced during practice is neither too high nor too low (Guadagnoli and Lee, 2004). Future research should also establish the test-retest reliability of this measurement approach for use in intervention and longitudinal study designs, and to facilitate translation to clinical practice.

## Ethics Statement

This perspective article includes data from a pilot study. The pilot study was carried out in accordance with the recommendations of the University of Florida Institutional Review Board with written informed consent from all subjects. All subjects gave written informed consent in accordance with the Declaration of Helsinki. The protocol was approved by the University of Florida Institutional Review Board and the North Florida/South Georgia Veterans Affairs Human Research Protection Program.

## Author Contributions

DC and DR: study design. DC, SC, and DO: data collection. SC and DC: data analysis. DC, EP, SC, DR, and DO: interpretation. SC, DC, DR, DO, and EP: preparation of the manuscript.

## Conflict of Interest Statement

The authors declare that the research was conducted in the absence of any commercial or financial relationships that could be construed as a potential conflict of interest.
